# CD147 Facilitates the Pathogenesis of Psoriasis through Glycolysis and H3K9me3 Modification in Keratinocytes

**DOI:** 10.34133/research.0167

**Published:** 2023-06-08

**Authors:** Chao Chen, Xiaoqing Yi, Panpan Liu, Jie Li, Bei Yan, Detian Zhang, Lei Zhu, Pian Yu, Lei Li, Jiaxiong Zhang, Yehong Kuang, Shuang Zhao, Wu Zhu, Cong Peng, Xiang Chen

**Affiliations:** ^1^ Department of Dermatology, Xiangya Hospital, Central South University, Changsha, Hunan, China.; ^2^ National Engineering Research Center of Personalized Diagnostic and Therapeutic Technology, Changsha, Hunan, China.; ^3^ Furong Laboratory, Changsha, Hunan, China.; ^4^ Hunan Key Laboratory of Skin Cancer and Psoriasis, Hunan Engineering Research Center of Skin Health and Disease, Xiangya Hospital, Central South University, Changsha, Hunan, China.; ^5^ National Clinical Research Center for Geriatric Disorders, Xiangya Hospital, Central South University, Changsha, Hunan, China.

## Abstract

Psoriasis is a chronic inflammatory skin disease featuring rapid proliferation of epidermal cells. Although elevated glycolysis flux has been reported in psoriasis, the molecular mechanisms underlying its pathogenesis remain unclear. We investigated the role of the integral membrane protein CD147 in psoriasis pathogenesis, observing its high expression in psoriatic skin lesions of humans and imiquimod (IMQ)-induced mouse models. In mouse models, genomic deletion of epidermal CD147 markedly attenuated IMQ-induced psoriatic inflammation. We found that CD147 interacted with glucose transporter 1 (Glut1). Depletion of CD147 in the epidermis blocked glucose uptake and glycolysis in vitro and in vivo. In CD147-knockout mice and keratinocytes, oxidative phosphorylation was increased in the epidermis, indicating CD147's pivotal role in glycolysis reprogramming during pathogenesis of psoriasis. Using non-targeted and targeted metabolic techniques, we found that epidermal deletion of CD147 significantly increased the production of carnitine and α-ketoglutaric acid (α-KG). Depletion of CD147 also increased transcriptional expression and activity of γ-butyrobetaine hydroxylase (γ-BBD/*BBOX1*), a crucial molecule for carnitine metabolism, by inhibiting histone trimethylations of H3K9. Our findings demonstrate that CD147 is critical in metabolic reprogramming through the α-KG–H3K9me3–*BBOX1* axis in the pathogenesis of psoriasis, indicating that epidermal CD147 is a promising target for psoriasis treatment.

## Introduction

Rapidly proliferating cells and cancer cells are characterized by remarkable alterations in their metabolism, particularly regarding their utilization of glucose. Even under sufficient oxygen conditions, rapidly proliferating cells catalyze glucose to produce lactate rather than metabolizing pyruvate for subsequent oxidative phosphorylation (OXPHOS) [1,2]. Owing to this metabolic alteration, mitochondrial-dependent OXPHOS is replaced by cytoplasmic anaerobic glycolysis to provide the necessary energy and macromolecular materials to meet metabolic and biosynthetic demands [2].

α-Ketoglutaric acid (α-KG) is a key metabolite in the mitochondrial tricarboxylic acid (TCA) cycle, serving both as a precursor in biosynthesis and as a co-factor in regulating distinct cellular effector functions [1]. For example, α-KG, produced by the deamination of glutamate, is a substrate of dioxygenases, which catalyze the removal of methyl marks from histones and control the activation of gene expression through epigenetic modifications [3,4]. α-KG is required for the activity of γ-butyrobetaine dioxygenase (γ-BBD or BBOX), which belongs to the 2-oxoglutarate (2OG/α-KG)-dependent dioxygenase superfamily and is encoded by the *BBOX1* gene [5]. γ-BBD catalyzes the formation of L-carnitine from γ-butyrobetaine (γ-BB) in the final step of the L-carnitine biosynthesis pathway [6]. BBOX1-mediated carnitine metabolism is thought to be negatively correlated with carcinogenesis, including hepatocellular carcinoma [7,8].

Psoriasis is a chronic inflammatory skin disease involving the hyperproliferation of keratinocytes (KCs), dermal cellular inflammatory infiltration, and angiogenesis; it is characterized by thickened, scaly plaques [9–11]. Metabolic alterations in the pathogenesis of psoriasis have been revealed by recent studies. In mouse models, imiquimod (IMQ)-induced psoriasis-like inflammation was suppressed by either genetically or pharmacologically blocking the expression of glucose transporter 1 (Glut1) or by inhibiting glycolysis by 2-deoxy-D-glucose (2-DG) [9,12], which indicated that highly expressed epidermal Glut1 elevates glycolysis activity and is critical in psoriasis.

CD147 (also known as basigin or Bsg) is an integral membrane protein of the immunoglobulin superfamily [13]. It is widely expressed in different cell types, including endothelial, epithelial, and immune cells [13]. CD147 exerts a variety of biological functions, including T-cell development, sperm development, and embryonic implantation [13–15]. CD147 is also ubiquitously expressed in circulating immune cell populations, such as activated T and B lymphocytes, dendritic cells, monocytes, and macrophages [16,17]. It is involved in inflammatory and immune diseases, including allergic asthma [18] and rheumatoid arthritis [19].

Our previous study showed that CD147 acts as a psoriasis susceptibility gene and is important in IL-22-mediated signaling pathways [20]. However, the role of epidermal CD147 in psoriasis remains elusive. We conducted a preclinical study on the role of epidermal CD147 in the pathogenesis of psoriasis and found that glycolysis flux and glucose uptake were distinctly upregulated in IMQ-induced psoriasis-like inflammation. Genomic epidermal knockout of CD147 markedly abrogated IMQ-triggered metabolic alterations. These results suggest that CD147 orchestrates metabolic alteration in KCs during the pathogenesis of psoriasis.

## Results

### Epidermal knockout of CD147 markedly attenuated IMQ-induced psoriasis-like dermatitis

Our previous study demonstrated that CD147 is a marker of high proliferation and poor differentiation in KCs and is believed to be a psoriasis susceptibility gene [21,22]. We observed remarkably increased expression of CD147 in non-immune cells (CD45^-^CD147^+^) and the accumulation of infiltrated inflammatory cells in skin lesions of patients with psoriasis compared with samples from healthy controls (Fig. S1A and B). There was a positive correlation between CD147 expression and the accumulation of inflammatory cells (Fig. S1C). We also found that CD147 was significantly highly expressed in IMQ-induced skin lesions (Fig. S1D).

**Fig. 1.**
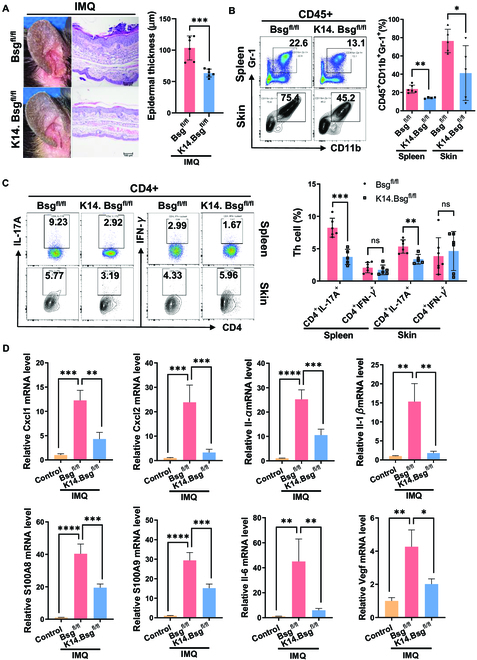
Epidermal knockout of CD147 markedly attenuated IMQ-induced psoriasis-like dermatitis. (A) Macroscopic views of ear skin with hematoxylin and eosin staining after IMQ induction (from *Bsg*^fl/fl^ and *K14.Bsg*^fl/fl^ mice). One representative mouse from each group is presented (*n* = 4 to 7 mice per group). Scale bars: 100 μm. The right panel shows statistics of epidermal thickness analysis. (B and C) Representative flow cytometry panels for quantification of CD45^+^CD11b^+^Gr-1^+^ cells after IMQ induction. (B) Th17 and Th1; (C) in spleens and skin lesions of *Bsg*^fl/fl^ and *K14.Bsg*^fl/fl^ mice (*n* = 4 to 7 mice per group). The right panel shows statistics of flow cytometric analysis. (D) Relative mRNA expression in the skin lesions of *Bsg*^fl/fl^ and *K14.Bsg*^fl/fl^ mice 2 days after IMQ induction (or without induction). The results were normalized to β-actin.

To further investigate the function of CD147 in psoriasis, mice with loxP-flanked CD147 alleles (*Bsg*^fl/fl^ mice) were crossed with keratin14-Cre mice (*K14.Bsg*^fl/fl^ mice) to knock out CD147 in KCs (Fig. S2A). Deletion of CD147 in the epidermis alleviated IMQ-induced psoriasis-like skin inflammation and significantly reduced epidermal thickening, compared with that of *Bsg*^fl/fl^ mice (Fig. 1A). We also observed significantly elevated CD147 mRNA expression in IMQ-induced skin lesions, but not in *K14.Bsg*^fl/fl^ mice (Fig. S2D). To further investigate the effect of epidermal CD147 on IMQ-induced inflammation, we conducted flow cytometry analysis. Deletion of CD147 in the epidermis led to a significant reduction in IMQ-induced accumulation of infiltrating CD45^+^CD11b^+^Gr-1^+^ inflammatory cells in skin lesions and the spleen (Fig. 1B). We examined the populations of Th17, Th1, and Treg cells in the spleen and skin lesions of *K14.Bsg*^fl/fl^ mice after IMQ treatment. The population of Th17 cells was dramatically decreased in the spleen and skin lesions, but the populations of Th1 cells did not change (Fig. 1C). Specific deletion of CD147 in the epidermis remarkably reduced the infiltration of Treg cells in the spleen (Fig. S2B).

Moreover, we measured the expression levels of several cytokines in skin lesions of *K14.Bsg*^fl/fl^ mice after treatment with IMQ for 48 h. The transcriptional expressions of *Cxcl1, Cxcl2, Il-1β, Il-1α, S100A8, S100A9, Il-6*, and *Vegf* were significantly decreased in skin lesions of *K14.Bsg*^fl/fl^ mice (Fig. 1D), indicating the proinflammatory role of the epidermal expression of CD147 in psoriasis.

### Epidermal deletion of CD147 abrogated glucose uptake and glycolytic capacity in IMQ-induced psoriatic dermatitis

**Fig. 2.**
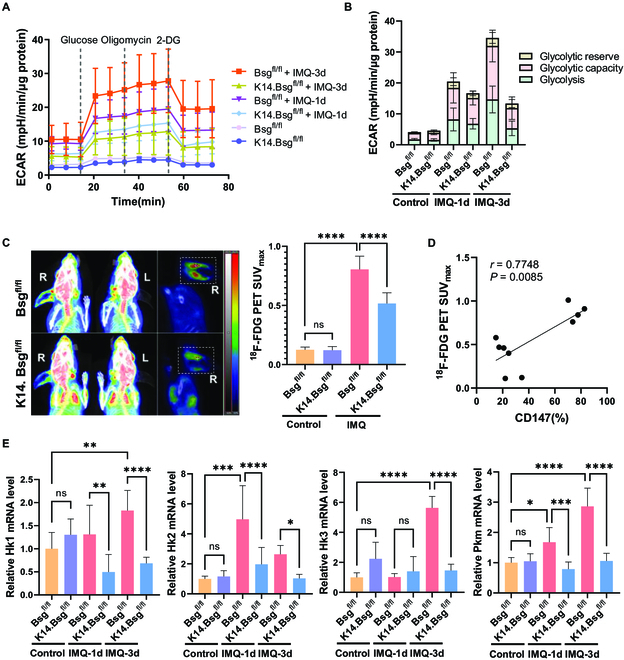
Epidermal deletion of CD147 abrogated glucose uptake and glycolytic capacity in IMQ-induced psoriatic dermatitis. (A) Real-time extracellular flux analysis of ECAR of full-skin cells derived from *Bsg*^fl/fl^ and *K14.Bsg*^fl/fl^ mice induced by IMQ for 0, 1, and 3 consecutive days (*n* = 4 to 6 mice per group). (B) Multiple stacked column chart of glycolysis, glycolysis capacity, and glycolysis reserve level. Statistical data are shown in Fig. S2E. (C) Representative ^18^F-FDG PET/CT imaging of *Bsg*^fl/fl^ and *K14.Bsg*^fl/fl^ mice induced by IMQ. The right panel shows the statistical analysis of ^18^F-FDG PET SUVmax (*n* = 4 to 6 mice per group). (D) The positive correlation between ^18^F-FDG PET SUVmax and CD147 (%). (E) Relative mRNA expression of *Hk1, Hk2, Hk3*, and *Pkm* in skin lesions of *Bsg*^fl/fl^ and *K14.Bsg*^fl/fl^ mice induced by IMQ for 0, 1, and 3 consecutive days.

Given that Glut1-mediated glucose uptake plays a key role in the growth of KCs in psoriasis [9], we proposed that glycolysis flux would be altered during psoriasis pathogenesis. As expected, the extracellular acidification rate (ECAR) [23], glycolysis, and glycolytic capacity were remarkably increased in full-skin lesions of IMQ-induced psoriatic dermatitis, while depletion of epidermal CD147 abrogated those alterations (Fig. 2A and B). We measured glucose uptake in IMQ-induced *K14.Bsg*^fl/fl^ and control mice through [^18^F]-fluoro-deoxyglucose positron emission tomography/computed tomography (^18^F-FDG PET-CT). ^18^F-FDG PET SUVmax was significantly higher in IMQ-induced mice than in controls (Fig. 2C), whereas epidermal knockout of CD147 remarkably reduced IMQ-induced ^18^F-FDG PET SUVmax (Fig. 2C). The value of ^18^F-FDG PET SUVmax was positively correlated with CD147 expression in skin lesions (Fig. 2D), indicating that CD147 regulates glycolysis flux through glucose uptake. In addition, the transcriptional expression of key glycolysis metabolic enzymes was reduced in IMQ-induced *K14.Bsg*^fl/fl^ mice compared with control mice, including hexokinases (*Hk*) 1, 2, and 3 and pyruvate kinase (*Pkm*) (Fig. 2E).

### CD147 played a critical role in the switch from OXPHOS to glycolysis in KCs

**Fig. 3.**
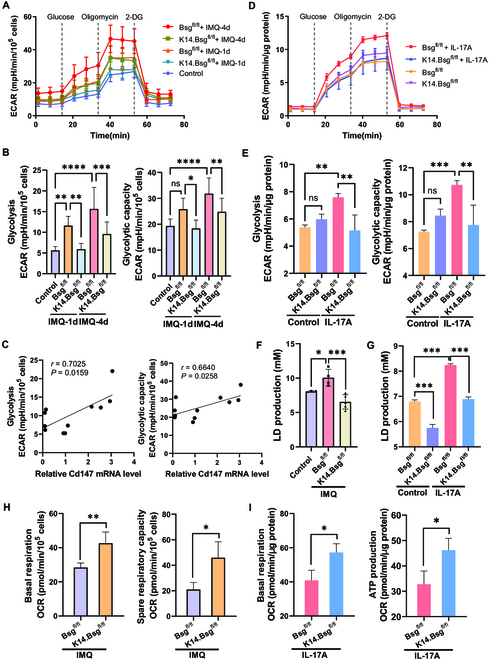
CD147 played a critical role in the switch from OXPHOS to glycolysis in KCs. (A) Real-time extracellular flux analysis of ECAR of KCs derived from the epidermis of *Bsg*^fl/fl^ and *K14.Bsg*^fl/fl^ mice after IMQ induction for 0, 1, and 4 consecutive days (*n* = 4 to 6 mice per group). (B) Statistical analysis of glycolysis and the glycolysis capacity level of KCs derived from the epidermis of IMQ-induced mice. (C) The positive correlation between glycolytic activity and relative mRNA expression of CD147. (D) Real-time extracellular flux analysis of ECAR of primary KCs derived from the epidermis of *Bsg*^fl/fl^ and *K14.Bsg*^fl/fl^ newborn mice after IL17A induction (100 ng/ml), along with mice not subjected to induction. (E) Statistical analysis of glycolysis and the glycolysis capacity level of IL17A-induced primary KCs. (F and G) Lactic acid production in plasma of *Bsg*^fl/fl^ and *K14.Bsg*^fl/fl^ mice induction by IMQ (F) or in supernatant of IL-17A-treated KCs (G). (H and I) Real-time extracellular flux analysis of mitochondrial OCR (reflecting oxidative phosphorylation) of KCs derived from the epidermis of *Bsg*^fl/fl^ and *K14.Bsg*^fl/fl^ mice after induction by IMQ (H) or IL17A (I). Statistical analyses of basal respiration, spare respiratory capacity, and ATP production.

To further investigate the role of epidermal CD147 in regulating the energy metabolism of KCs in psoriasis, we conducted glycolysis stress tests in the KCs of *K14.Bsg*^fl/fl^ transgenic mice treated with IMQ or IL-17A. Consistent with our aforementioned results, the ECAR, glycolysis, and glycolytic capacity were significantly increased in the epidermis, while the deletion of epidermal CD147 inhibited this metabolic phenotype (Fig. 3A and B). Both ECAR of glycolysis and glycolysis capacity were positively correlated with relative CD147 transcriptional expression in the epidermis (Fig. 3C).

IL-17A is an inflammatory cytokine linked to psoriasis development and severity; using anti-IL-17 antibodies to block it is an effective existing treatment in psoriasis therapy [24]. IL-17A targets KC activation, which leads to dysfunction through the p38-MAPK and NF-κB signaling pathways [25,26]. We treated mouse primary KCs with IL-17A and observed that the glycolysis capacity was significantly elevated. Knockout of CD147 in KCs abrogated IL-17A-induced glycolytic activity (Fig. 3D and E).

CD147 has also been reported to regulate lactate production. In line with our previous results, knockout of CD147 in the epidermis remarkably reduced lactate production in the plasma of IMQ-induced psoriatic mice (Fig. 3F) and in the supernatant of IL-17A-treated KCs isolated from *K14.Bsg*^fl/fl^ mice (Fig. 3G). Knockout of CD147 in the epidermis dramatically raised the basal mitochondrial oxygen consumption rate (OCR) and spare respiratory capacity (maximal OCR–basal OCR) in the epidermis of IMQ-induced psoriatic mice (Fig. 3H). The basal mitochondrial OCR and the OCR of adenosine triphosphate (ATP) production were also significantly upregulated in CD147 knockout KCs after treatment with IL-17A (Fig. 3I). These results suggest that epidermal CD147 is critical in the reprogramming of the metabolism of psoriatic KCs away from OXPHOS and toward glycolysis.

### Epidermal CD147 facilitated glucose uptake through Glut1 in KCs

**Fig. 4.**
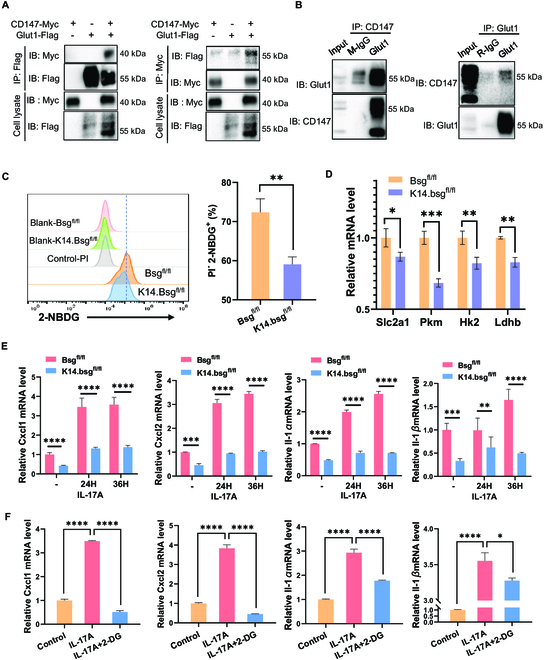
Epidermal CD147 facilitated glucose uptake through Glut1 in KCs. (A) CD147 binds to Glut1: 293T cells were co-transfected with CD147-Myc and Glut1-Flag plasmids. Co-immunoprecipitation was performed using anti-Myc or anti-Flag antibodies, followed by immunoblotting with the indicated antibodies. (B) CD147 binds to endogenous Glut1: HaCaT cell extracts were used for immunoprecipitation with anti-CD147/Glut1 antibody or immunoglobulin G as a control. The complex was detected by immunoblotting with anti-Glut1/CD147 antibody. (C) Representative flow cytometry panels for 2-NBDG uptake in primary KCs derived from *Bsg*^fl/fl^ and *K14.Bsg*^fl/fl^ mice. The right panel shows statistical analysis of PI^-^2-NBDG^+^ cells (%). (D) Relative mRNA expression of *Slc2a1*, *Pkm, Hk2*, and *Ldhb* in primary KCs derived from *Bsg*^fl/fl^ and *K14.Bsg*^fl/fl^ mice. (E) Relative mRNA expression of Cxcl1/2 and IL-1α/β in primary KCs induced by IL-17A (100 ng/ml) for 0, 24, and 36 h. (F) Relative mRNA expression of Cxcl1/2 and Il-1α/β in primary KCs induced by IL-17A (100 ng/ml) or IL-17A + 2-DG (10 mM) for 12 h.

Enhanced glucose uptake mediated by Glut1 is a key step in the Warburg effect, leading to an elevated rate of anaerobic glycolysis and a reduced rate of OXPHOS [1,27]. We co-transfected the plasmids CD147-Myc and SLC2A1-Flag into 293T cells and performed immunoprecipitation assays using anti-c-Myc or anti-Flag antibodies. CD147-Myc and SLC2A1-Flag were observed within the immunoprecipitated complex (Fig. 4A). Glut1 expression was detected in the anti-CD147 antibody immunoprecipitated complex in HaCaT cells but not in the immunoglobulin G (IgG) control antibody group, and vice versa (Fig. 4B), indicating that Glut1 is a novel interacting protein of CD147 in KCs. Furthermore, we also detected the co-localization of CD147 and GLUT1 on the cell membrane of psoriasis skin lesions and HaCaT cells by using laser confocal microscopy (Fig. S1E and F).

Moreover, the deletion of CD147 in primary KCs significantly decreased intracellular glucose uptake in vitro, using the fluorescent glucose analog 2-(N-(7-nitrobenz-2-oxa-1,3-diazol-4-yl) amino)-2-de-oxyglucose (2-NBDG) uptake assay (Fig. 4C). Transcriptional expression of solute carrier family 2 member 1 (*Slc2a1*), pyruvate kinase (*Pkm*), hexokinase 2 (*Hk2*), and lactate dehydrogenase B (*Ldhb*) were suppressed in primary KCs depleted of epidermal CD147 (Fig. 4D). We confirmed that deletion of CD147 in KCs (Fig. 4E) and pharmacological inhibition of glycolysis by 2-DG (Fig. 4F) attenuated the expression of IL-17A-induced proinflammatory molecules, including *Cxcl1*, *Cxcl2*, *IL-1α*, and *IL-1β*, indicating that increased glycolytic activity aggravated the production of inflammatory factors in KCs.

### Epidermal depletion of CD147 enhanced carnitine metabolism through a combination of metabolomics and transcriptomics analysis in KCs

**Fig. 5.**
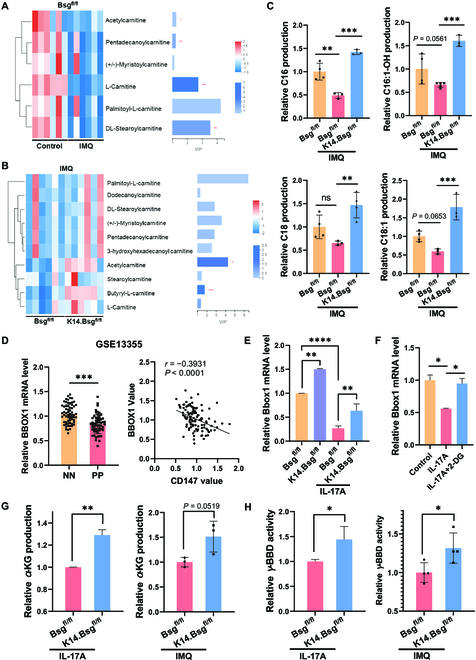
Epidermal depletion of CD147 raised carnitine metabolism through a combination of metabolomics and transcriptomics analysis in KCs. (A and B) Heatmap showing the differential abundance of carnitine and acylcarnitines (A) between the *Bsg*^fl/fl^ control group and *Bsg*^fl/fl^/IMQ group and (B) between the *Bsg*^fl/fl^/IMQ group and the *K14.Bsg*^fl/fl^/IMQ group (*n* = 6 mice per group). (C) The relative concentration distribution of differential acylcarnitines in IMQ-induced mouse models among the 3 groups derived by carnitine-targeted metabolomics profiling analysis (*n* = 3 to 5 mice per group). (D) Relative mRNA expression of *Bbox1* (left) and the negative correlation between *BBOX1* and CD147 expression (right) in full skin of patients with psoriasis from public database GSE13355. (NN, healthy controls; PP, patients with psoriasis.) (E) Relative mRNA expression of *Bbox1* in primary KCs derived from *Bsg*^fl/fl^ and *K14.Bsg*^fl/fl^ mice after induction by IL-17A (100 ng/ml) for 12 h. (F) Relative mRNA expression of *Bbox1* in primary mice KCs after induction by IL-17A (100 ng/ml) or IL-17A + 2-DG (10 mM) for 12 h. (G and H) Relative α-KG production (G) and γ-BBD activity (H) in KCs derived from the epidermis of *Bsg*^fl/fl^ and *K14.Bsg*^fl/fl^ mice after induction by IMQ for 3 days or IL-17A for 36 h.

To further investigate the effect of CD147 on metabolic profiles in psoriasis, we employed a non-targeted metabolomics approach to the epidermis of IMQ-induced psoriatic dermatitis in *K14.Bsg*^fl/fl^ mice (Fig. S3). Compared with the control group, IMQ treatment dramatically reduced carnitine metabolism. In contrast, carnitine metabolism was dramatically increased in the epidermis of IMQ-induced *K14.Bsg*^fl/fl^ mice (Fig. 5A and B). Carnitine-targeted metabolomic profiling further validated that depletion of epidermal CD147 abrogated IMQ-induced inhibition of carnitine production for Dodecenoylcarnitine (C12:1), Tetradecanoylcarnitine (C14), Hexadecanoylcarnitine (C16), Hexadecenoylcarnitine (C16:1), Hydroxyhexadecenoylcarnitine (C16:1-OH), Octadecanoylcarnitine (C18), and Octadecenoylcarnitine (C18:1) (Fig. 5C and Fig. S4).

To further study the effect of CD147 on psoriasis, we performed RNA-seq analysis of transcriptional alteration in KCs isolated from *K14.Bsg*^fl/fl^ mice after IMQ treatment for 3 consecutive days. Specifically, Kyoto Encyclopedia of Genes and Genomes pathway analysis determined that the most important differential expressions of enriched pathways included biosynthesis of unsaturated fatty acids, fatty acid elongation, and fatty acid metabolism (Fig. S5A). We subsequently performed gene set enrichment analysis, which showed that the differentially expressed genes were enriched in cytokine–cytokine receptor interaction, chemokine IL-17, and tumor necrosis factor (TNF) signaling pathways (Fig. S5B). The expression of the carnitine metabolism-related gene *Bbox1* was significantly elevated in the epidermis of IMQ-induced *K14.Bsg*^fl/fl^ mice (Fig. S5C and D). *BBOX1* encodes γ-BBD, which synthesizes endogenous carnitine by the hydroxylation of γ-BB [5,6]. We also found that *BBOX1* was downregulated in skin lesions of patients with psoriasis, based on the analysis of a public database, and *BBOX1* was negatively related to CD147 expression in psoriasis skin lesions (Fig. 5D), indicating that CD147 has an essential role in carnitine biosynthesis in psoriasis.

We observed that the transcriptional expression of *Bbox1* was downregulated in IL-17A-stimulated mouse primary KCs, while either depletion of CD147 or treatment with 2-DG revived the expression of *Bbox1* (Fig. 5E and F). Depletion of CD147 markedly increases TCA activity, as described in our previous results. Therefore, we tested the key metabolites in the TCA cycle and found that the production of α-KG was upregulated in IL-17A-treated primary KCs with CD147 deletion and in the epidermis of IMQ-induced *K14.Bsg*^fl/fl^ mice (Fig. 5G). Given that γ-BBD is an α-KG/Fe^2+^-dependent dioxygenase, with α-KG acting as a co-factor in their enzymatic activities, we further examined whether the deletion of epidermal CD147 affects γ-BBD activity. As shown in Fig. 5H, genomic deletion of CD147 significantly elevated γ-BBD activity in IL-17A-treated primary KCs as well as in the epidermis of IMQ-induced psoriatic mice.

### CD147 regulated BBOX1 expression through H3K9me3 in KCs

**Fig. 6.**
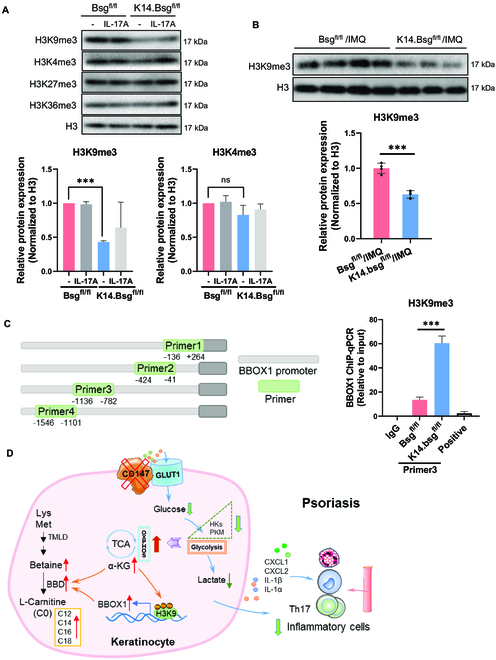
CD147 regulates BBOX1 expression through H3K9me3 in KCs. (A) Representative immunoblot bands and histogram of relative expression of H3K9me3 in primary KCs derived from the epidermises of *Bsg*^fl/fl^ and *K14.Bsg*^fl/fl^ mice after induction by IL-17A (100 ng/ml) for 36 h, as well as those without induction. (B) Representative immunoblot bands and histogram of relative expression of H3K9me3 in the epidermis of *Bsg*^fl/fl^ and *K14.Bsg*^fl/fl^ mice after induction by IMQ for 3 days. (C) The association of H3K9me3 with Bbox1 was assessed using ChIP assays. The DNA was immunoprecipitated with the specific antibody H3K9me3. Bars represent the relative levels of the PCR product of the *Bbox1* promoter region’s association with H3K9me3. (D) Schematic illustration of the CD147/Glut1/α-KG/H3K9me3/*Bbox1* pathways contributing to the pathogenesis of psoriasis.

α-KG is a well-known critical co-factor for the Jumonji domain-containing histone demethylases that catalyze the removal of methyl marks from histones [3,4]. Given that the level of α-KG was elevated in CD147-knockout KCs and in mice with psoriatic dermatitis, we proposed that CD147 regulates histone modifications. Therefore, we further examined the histone methylation expressions of H3K9me3, H3K4me3, H3K27me3, and H3K36me3, which have been documented to be induced by α-KG for histone modification. We found that depletion of CD147 blocked H3K9me3 expression in primary KCs derived from *Bsg*^fl/fl^ or *K14.Bsg*^fl/fl^ mice (Fig. 6A), as well as in epidermis isolated from IMQ-treated *Bsg*^fl/fl^ or *K14.Bsg*^fl/fl^ mice (Fig. 6B). γ-BBD belongs to the 2OG/α-KG-dependent dioxygenase superfamily of enzymes and is encoded by the *BBOX1* gene [5]. It catalyzes the last step of the L-carnitine biosynthesis pathway [6]. As reported in the above result (Fig. 5E), we found that CD147 knockout remarkably increased the transcriptional expression of *Bbox1.* Therefore, we speculated that CD147 regulates *Bbox1* expression through H3K9me3. We used a public database to analyze the H3K9me3 expression profile in the *Bbox1* promoter area, and then we designed the primers for chromatin immunoprecipitation (ChIP) assays (Table S3). As expected, the expression of H3K9me3 in the *Bbox1* promoter area was markedly enhanced in KCs isolated from *K14.Bsg*^fl/fl^ mice (Fig. 6C). This indicated that epidermal CD147 regulates *Bbox1* expression through H3K9me3. Our findings revealed that CD147 has a critical role in metabolic reprogramming through the α-KG–H3K9me3–*BBOX1* axis in the pathogenesis of psoriasis, indicating that epidermal CD147 is a potentially promising target molecule for psoriasis treatment (Fig. 6D).

## Discussion

Elevated glucose uptake (regulated by Glut1) is believed to be critical in the metabolic reprogramming of rapidly proliferating cells [9,27,28]. A high expression level of Glut1 is well documented in most malignant tumor cells. Glut1 promotes the ability of cells to utilize and accelerate the catabolism of glucose, which increases the supply of energy and biomacromolecules for maintaining the malignant phenotype [2,28–31]. Accumulating evidence shows that Glut1 is overexpressed in psoriatic skin lesions [9,12]. Furthermore, PET/CT imaging reveals that cutaneous ^18^FDG uptake corresponds to clinically apparent psoriatic lesions [32]. Our results also reveal that glycolytic capacity was dramatically increased in both the epidermis and full skin of IMQ-induced psoriatic skin lesions. Most importantly, the rate of ^18^FDG uptake was upregulated in skin lesions of IMQ-induced psoriasis-like mouse models, suggesting high consumption of glucose in the process of psoriasis.

CD147 is a member of the immunoglobulin superfamily, which has a variety of biological functions, including spermatogenesis, T-cell maturation, and carcinogenesis [13–15,33,34]. Particularly, CD147 is critical in tumor glycolysis [28,35]. It has been reported that CD147 partners with either monocarboxylate transporter (MCT)1 or MCT4 for the transmembrane transport of lactate [14,36]. Deletion or disruption of CD147 is associated with a break in MCT-mediated lactate transport in tumor cells [37–39]. In this study, we showed that CD147 is a novel partner with Glut1 in KCs. The deletion of epidermal CD147 alleviated IMQ-induced glucose uptake, lactate production, and psoriasis-like dermatitis. The mitochondrial OCR was distinctly increased in CD147 epidermal knockout mice, which suggests that the depletion of CD147 reverses TCA capacity. Our results also show that IL-17A (a critical proinflammatory cytokine in the pathogenesis of psoriasis) induced increased glycolysis, lactate production, and psoriasis-related factors, including the expression of CXCL1/2 and IL-1α/β, in mouse primary KCs. Depletion of CD147 or blocking glycolysis with a pharmacological inhibitor markedly suppressed the expression of those IL-17A-induced factors, indicating that elevation of glycolysis flux is required for KCs to produce proinflammatory factors in psoriasis.

CD147 is also known to be involved in the regulation of amino acid and fatty acid metabolism [28,35,40,41]. Unexpectedly, we found that depletion of CD147 in the epidermis elevated acyl-carnitine metabolism, including C12, C14, C16, and C18, which were suppressed in IMQ-induced psoriasis-like inflammation. We previously found 14 significantly downregulated carnitines, including C16, in the plasma of patients with psoriasis, suggesting a depressed state of fatty acid β-oxidation [42]. We also found that carnitine supplementation markedly reduced IMQ-induced epidermal thickening and infiltration of Th17 cells in skin lesions [42]. Carnitine has an essential metabolic role in transporting fatty acids into the mitochondria for β-oxidation [43,44]. The endogenous carnitine pool is composed of carnitine and various acylcarnitines, and the inhibition of carnitine metabolism reduces fatty acid oxidation [44]. In humans, 75% of carnitine is obtained from the diet, while 25% relies on autologous biosynthesis [45]. A prospective cohort study among UK Biobank participants of European ancestry indicated that the effect of diet was with smaller effect sizes [46]. Due to the complexity of carnitine and fatty acid metabolism in vivo, the relationship between psoriasis and carnitine and fatty acid metabolism still needs further studies to be confirmed. In this study, we found that the expression of the carnitine metabolism-related gene *BBOX1* was significantly elevated in the epidermis of IMQ-induced *K14.Bsg*^fl/fl^ mice (Fig. S5). *BBOX1* was negatively related to CD147 expression in psoriasis skin lesions (Fig. 5D), indicating that CD147 has an essential role in carnitine biosynthesis in psoriasis.

Epidermal knockout of CD147 increased the capacity of TCAs and the abundance of α-KG. The TCA cycle is a central metabolic pathway within mitochondria; the TCA cycle flux promotes biosynthesis and regulates chronic inflammatory diseases. For example, a perturbed TCA cycle metabolism enhances the production of mitochondrial reactive oxygen species in dendritic cells and promotes IL-23 expression and skin inflammation [47]. Upregulation of the TCA cycle enhances oxidative stress responses that aggravate inflammatory reactions in atopic dermatitis [48]. Increased succinate in the synovial fluid of patients with rheumatoid arthritis induces macrophages to release IL-1β, promoting inflammation [49]. α-KG is an important intermediate in the TCA cycle, classified as an anti-inflammatory phenotype in adipose tissue, thrombosis inflammation, and colitis [50–52]. Mechanistically, α-KG promotes DNA demethylation in adipocytes, mediated by ten-eleven translocation enzymes, and attenuates STAT3/NF-κB signaling by its receptor, oxoglutarate receptor 1 [50]. α-KG inhibits the inflammation of thrombosis through phospho-Akt inactivation mediated by prolyl hydroxylase-2 [51]. α-KG alleviates colitis by regulating stem cell proliferation through Wnt–Hippo signaling [52].

Histone modifications have been reported in the pathogenesis of psoriasis [53]. The downregulation of H3K9 dimethylation was shown to be clinically relevant to IL-23 expression in psoriatic skin lesions [54], while H3K27me3 and EZH2 (a histone methyltransferase) were substantially enriched in psoriatic lesions [55]. In psoriatic peripheral blood mononuclear cells, there were reduced levels of acetylated H3 and H4, and increased levels of methylated H3K4, which were associated with a biological drug response [56]. H3K9me3 is a well-known epigenetic hallmark of heterochromatin status (compacted, transcriptionally repressed chromatin), which represses gene expression in pathological and physiological processes [57,58]. H3K9me3 has been reported to be involved in glycolysis and the expression of proinflammatory factors. Increased levels of H3K9me3 have been observed to repress the expression of glucose-6-phosphate dehydrogenase promoter, reducing the level of tumor-reactive cytotoxic T lymphocytes [59], and facilitating the growth of human mesothelioma cells [60]. Furthermore, decreased H3K9me3 level increased the levels of Hk2, TNF-α, IL-6, and PFKP, which benefited glycolysis activity and the expression of proinflammatory factors during Bacillus Calmette–Guérin vaccine-induced trained immunity [61]. Furthermore, evidence demonstrates that other proinflammatory factors are mediated by H3K9me3, including IL-17A [62], IL-1β [63,64], and CXCL1 [65]. Our study detected H3K9me3 in the promoter sites of *BBOX1*, an essential molecule for endogenous carnitine biosynthesis. This indicates that H3K9me3 may exert functions in carnitine metabolism; however, detailed future investigations are required.

In conclusion, we found that genomic deletion of CD147 in the epidermis suppressed the glycolytic rate via Glut1-mediated glucose uptake, resulting in increased TCA activity and a subsequent increase in a-KG production, observed in the epidermis of CD147-knockout mice and in mouse primary KCs. Increased γ-BBD activity and its transcriptional expression through H3K9me3 indicate that epidermal CD147 is a novel target for psoriasis therapy.

## Materials and Methods

### Study design

Mice with loxP-flanked CD147 alleles (*Bsg*^fl/fl^ mice) were crossed with keratin14-Cre mice (*K14.Bsg*^fl/fl^ mice) to knock out CD147 in KCs. The resulting skin phenotype was induced by IMQ and characterized by measuring the thickness of the epidermis and flow cytometric evaluation of infiltrating immune cells. For the in vivo experiments, ^18^F-FDG PET-CT was used to evaluate glucose uptake, RNA-seq technique was used to screen for differential genes and pathways, and non-targeted and targeted metabolic techniques were used to screen for differential metabolites. All experiments used newborn or 7- to 9-week-old male and female mice. For the in vitro experiments, KCs were isolated from newborn mice, cultured, and stimulated with IL-17A, and gene expression analysis and glucose uptake determination were performed.

### Human skin samples

This study was reviewed and approved by the local ethics Institutional Review Board (IRB; Xiangya Hospital, Central South University, IRB-201512526). All experiments were conducted in accordance with the principles of the Declaration of Helsinki. We collected skin samples from 45 individuals (18 patients with psoriasis vulgaris and 27 healthy controls). Table S1 summarizes the demographic characteristics of the subjects. Inclusion criteria included newly diagnosed and untreated patients with psoriasis and without any other inflammatory skin diseases. Patients with psoriasis and healthy control subjects were older than 18 years, gave written informed consent, and provided skin samples. Exclusion criteria included the use of subcutaneous and intravenous systemic immunosuppressive drugs. Clinical evaluation of the psoriasis subtype and Psoriasis Area and Severity Index (PASI) score was performed.

### Mice and treatments

Transgenic mice with a specific epidermis CD147 knockout (*K14.Bsg*^fl/fl^ mice) were generated by Shanghai Biomodel Organism Science and Technology Development Co. (Shanghai, China). The specific construction and genotyping methods have been previously reported [66].

CD147 transgenic mice did not show any pathogenic skin phenotype for at least 6 months. Mice were reproduced and maintained under specific pathogen-free conditions and provided with adequate food and water. According to the National Institutes of Health *Guide for the Care and Use of Laboratory Animals*, the grouping of experimental mice followed the principles of age and gender matching. The animal study protocol was approved by the Ethics Committee of Xiangya Hospital (Central South University, China, #2015110134).

The transgenic mice aged 6 to 8 weeks were smeared with either 62.5 mg of 5% IMQ cream (MedShine, cat.120503, China) on their backs or 20 mg of 5% IMQ cream on each ear once per day, and their skin lesions were observed and recorded once daily. A scoring system based on the clinical PASI was used to evaluate the skin inflammation on the skin lesions of mice. The mouse skin samples were collected and immediately fixed in a 4% paraformaldehyde solution (Servicebio, cat. G1101, China) for hematoxylin and eosin staining.

### Primary KC culture

Mouse primary KCs were isolated from *K14.Bsg^fl/fl^* newborn mice. The isolated skin was digested in either 1 ml of DPBS containing 5 mg/ml dispase II (Sigma-Aldrich, cat. D4693, USA) at 37 °C for 60 min or 2 mg/ml dispase II at 4 °C for 18 h. A 2- to 5-mm-long tail piece from each mouse was collected for genotyping. Then, the epidermis was torn off using tweezers. Next, the epidermis was digested in 1 ml of 0.25% Trypsin-EDTA Solution (Beyotime, cat. C0201) at 37 °C for 5 to 10 min. After this, 1 to 2 ml of Dulbecco's Modified Eagle Medium (DMEM) containing 10% fetal bovine serum was added to each dish. The KCs were obtained by centrifugation (400 × *g* at room temperature for 3 min) and cultured using Keratinocyte Growth Medium 2 (cat. C-20011; PromoCell, St. Louis, MO, USA).

### Tissue processing and flow cytometry

Mice tissue processing and flow cytometry were performed according to previously described methods [42]. Human skin samples were collected from Outpatient Clinic (about 3*3 mm/samples). Skin samples were cut into small pieces and digested in 3 ml of DMEM containing 2 mg/ml collagenase type IV (Sigma-Aldrich, cat. V900893, USA) and 100 μg/ml DNase I (Sigma-Aldrich, DN25, USA) while shaking at 37 °C for 60 to 90 min. Enzyme activity was stopped using 10% fetal bovine serum DMEM medium. The tissue was further homogenized with a syringe and filtered through a 70-μm cell strainer. The cell strainer was washed with 20 ml of PBS followed by centrifugation (500 × *g* at 4 °C for 5 min). Single cells were then stained with fluorescence antibodies (CD45, CD11b, CD33, and CD147) for flow cytometry. All antibodies used for flow cytometry are summarized in Table S4.

### IMQ-induced mice models and micro-PET/CT

The right ear of each *Bsg*^fl/fl^ mouse and *K14.Bsg*^fl/fl^ mouse was treated with 20 mg IMQ per day for 6 consecutive days, while the left ear was treated with vehicle control. After the establishment of IMQ-induced mouse models, 0.2 ml of ^18^F-FDG (200 μ Ci) was injected into the tail vein. Then, the mice were scanned using a Siemens Inveon micro-PET/CT. The images were analyzed using Inveon Acquisition Workplace [67], and the region of interest was obtained. Finally, the standard uptake value (SUV) of the region of interest (^18^F-FDG PET SUVmax) was calculated.

### Glucose uptake and lactate production assay

Glucose absorption was analyzed using a BD Fortessa multi-dimensional high-definition flow cytometer and a Glucose Uptake Cell-Based Assay Kit (No. 600470; Cayman Chemical Company, Ann Arbor, MI, USA). Lactate production was determined using a lactate concentration determination kit (Jiancheng, Nanjing, China) according to the manufacturer's protocol.

### Seahorse analytics

Mouse primary KCs were exposed to IL-17A for 36 h in vitro in a culture medium containing a physiological glucose concentration (6 mM) measured by the Seahorse XF Glycolysis and Cell Mito Stress Test Kit. The ECAR and the OCR were determined using a Seahorse XF96 Bioanalyzer (Seahorse Bioscience, North Billerica, MA, USA) according to the manufacturer's protocol.

### Untargeted metabolomics

The specific procedures for untargeted metabolomics were in accordance with a previously published study [68]. Briefly, 50 mg of mouse skin was collected and ground in a 2-ml centrifuge tube. An extract (methanol:water = 4:1 (v:v); 400 μl) containing 0.02 mg/ml internal standard (L-2-chlorophenylalanine) was added for the extraction of metabolites. The sample solution was ground in a frozen tissue grinder for 6 min (−10 °C, 50 Hz) and extracted by ultrasound at a low temperature for 30 min (5 °C, 40 kHz). The sample was then placed at −20 °C for 30 min and centrifuged for 15 min (4 °C, 13,000 × *g*), after which the supernatant was transferred to an injection vial with an endotracheal tube for computer analysis. All the metabolites were leveled to the same sample volume to prepare quality (QC) control samples. During instrument analysis, one QC sample was inserted for every 10 samples to check the repeatability of the entire analysis process. The instrument platform used for LC-MS analysis was the ultra-high performance liquid chromatography-tandem Fourier transform mass spectrometry UHPLC-Q Executive system from Thermo Fisher Scientific (Waltham, MA, USA). The data were analyzed using the software Majorbio Cloud Platform (www.majorbio.com).

### Acyl-carnitine quantification

The carnitine and acyl-carnitine in the mouse epidermis were measured using ultra-performance liquid chromatography-tandem mass spectrometry (UPLC-MS/MS). Each mouse epidermis sample was weighed (25 mg), 1 ml of ultrapure water was used for ultrasonic homogenization, and 2 μl was collected for the pretreatment experiment. Samples were analyzed using an AB SCIEX 3200MD QTRAP LC/MS/MS System (AB Sciex, USA) equipped with a Waters UPLC (Waters, Milford, MA, USA).

### Co-immunoprecipitation

The whole cell protein extract was lysed in NP40 buffer on ice for 30 min, and the lysate was centrifuged at 12,000 rpm for 10 min. The protein concentration was measured using the BCA protein assay kit (BioTeke Corporation). Protein (1 mg) and agarose beads (20 μl) were incubated at 4 °C for 1 h and centrifuged at 3,000 rpm for 3 min to collect the supernatant. Then, 1.5 μg of a specific antibody (#12939, Cell Signaling Technology [CST]; sc-21746, Santa Cruz) was added to the test tube and it was incubated overnight. Next, 40 μl of protein A/G agarose beads (Beyotime Biotechnology) were rotated at 4 °C for 2 h and then centrifuged at 3,000 rpm for 3 min to collect the agarose beads. All beads were then recycled and washed 3 times with 1 ml of NP40 buffer solution. Then, the columns were collected for protein denaturation and immunoblotting.

### α-KG level and γ-BBD activity evaluation

Intracellular αKG levels and intracellular γ-BBD activity in KCs and the epidermis were measured by using an α-KG assay kit (MAK054, Sigma-Aldrich) and mouse γ-BBD ELISA kit (LY30951-A, LVYE BIOTECHNOLOGY), respectively, according to the manufacturer's instructions.

### ChIP assay

The ChIP assay was performed using a SimpleChIP® Enzymatic Chromatin IP Kit (Magnetic Beads) (#9003, Cell Signaling Technology [CST]) following the manufacturer's protocol. The antibodies for the ChIP assay were H3K9me3 (#13969, CST, 2 μg/test) and IgG (supplied in the kit, 2 μg/test). The quantitative real-time PCR (qRT-PCR) primers of the *Bbox1* promoter used in the ChIP assay are listed in Table S3.

### Protein preparation and immunoblotting

Histones were extracted from KCs and the epidermis using a histone extraction kit (No. 40028, Active Motif) according to the manufacturer's instructions. Proteins were loaded on 10% SDS-polyacrylamide gel electrophoresis (PAGE) gels, transferred to polyvinylidene fluoride membranes (Millipore, USA), and visualized by Western blotting using the following specific antibodies: anti-H3K9me3 (1:1,000; #13969, CST), anti-H3K4me3 (1:1,000; #9751, CST), anti-H3K27me3 (1:1,000; #9733, CST), anti-H3K36me3 (1:1,000; #9763, CST), and anti-histone H3 (1:1,000; #4499, CST). The blots were imaged using a gel image analysis system (Bio-Rad, Hercules, CA, USA).

### qRT-PCR

Total RNA was extracted using MagZol reagent (Magen, #R4801-01), and cDNA was synthesized via reverse transcription using a HiScript Q RT Kit (Yeasen, #11141ES60). Then, qRT-PCR was performed using a 2× SYBR Green Qpcr Master mix with low ROX (Bimake, China) according to the manufacturer’s instructions on a QuantStudio 3 RT-PCR instrument (Thermo Fisher Scientific). The reaction mixture contained 0.5 ml of forward and reverse mouse primers, as described in Table S2. Values were normalized to β-actin.

### RNA-Seq

The cDNA library construction, library purification, and transcriptome sequencing were implemented according to BGI-Shenzhen Company's instructions.

### Statistical analysis

All statistical analyses were performed using GraphPad Prism 9 (GraphPad Software, San Diego, CA, USA). When the sample was not normally distributed, the statistical significance between the values was determined using either a 2-tailed unpaired Student's *t*-test or one-way ANOVA with Dunnett's post hoc test. The correlation between the measured variables was tested by Spearman rank correlation analysis. All data are presented as the mean ± SD. *P < 0.05; **P < 0.01; ***P < 0.001; ****P < 0.0001; ns, not significant.

## Data Availability

The data that support the findings of this study have been deposited into the CNGB Sequence Archive (CNSA) [69] of the China National GeneBank DataBase (CNGBdb) with accession number CNP0004096.

## Supplementary Materials

Supplementary 1 Fig. S1. The abundance of epidermal CD147 in skin lesions of patients with psoriasis is related to inflammatory cell infiltration. Fig. S2. Depleting CD147 attenuates IMQ-induced psoriasis-like skin inflammation and down-regulates inflammatory cell infiltration. Fig. S3.Non-targeted metabolomics profiling analysis for epidermis of Bsg^fl/fl^ and K14.Bsg^fl/fl^ mice. Fig. S4. Carnitine-targeted metabolomics profiling analysis for epidermis of Bsg^fl/fl^ and K14.Bsg^fl/fl^ mice. Fig. S5. The effect of depletion of CD147 on gene expression profiles for epidermis of Bsg^fl/fl^ and K14.Bsg^fl/fl^ mice treated with IMQ. Table S1. Demographics of patients with psoriasis and healthy control subjects. Table S2. A list of primers used for qPCR. Table S3. A list of primers for used ChIP-qPCR. Table S4. A list of antibodies used for flow cytometry.
