# Supplementary material for: CD147 Facilitates the Pathogenesis of Psoriasis through Glycolysis and H3K9me3 Modification in Keratinocytes
Source: Research (Wash D C). 2023 Jun 8;6:0167. doi: 10.34133/research.0167 (PMC10249783; doi:10.34133/research.0167)
Supplement: Supplementary 1 — Fig. S1. The abundance of epidermal CD147 in skin lesions of patients with psoriasis is related to inflammatory cell infiltration. Fig. S2. Depleting CD147 attenuates IMQ-induced psoriasis-like skin inflammation and down-regulates inflammatory cell infiltration. Fig. S3.Non-targeted metabolomics profiling analysis for epidermis of Bsgfl/fl and K14.Bsgfl/fl mice. Fig. S4. Carnitine-targeted metabolomics profiling analysis for epidermis of Bsgfl/fl and K14.Bsgfl/fl mice. Fig. S5. The effect of depletion of CD147 on gene expression profiles for epidermis of Bsgfl/fl and K14.Bsgfl/fl mice treated with IMQ. Table S1. Demographics of patients with psoriasis and healthy control subjects. Table S2. A list of primers used for qPCR. Table S3. A list of primers for used ChIP-qPCR. Table S4. A list of antibodies used for flow cytometry. [file research.0167.f1.docx]

**Supplementary Materials**


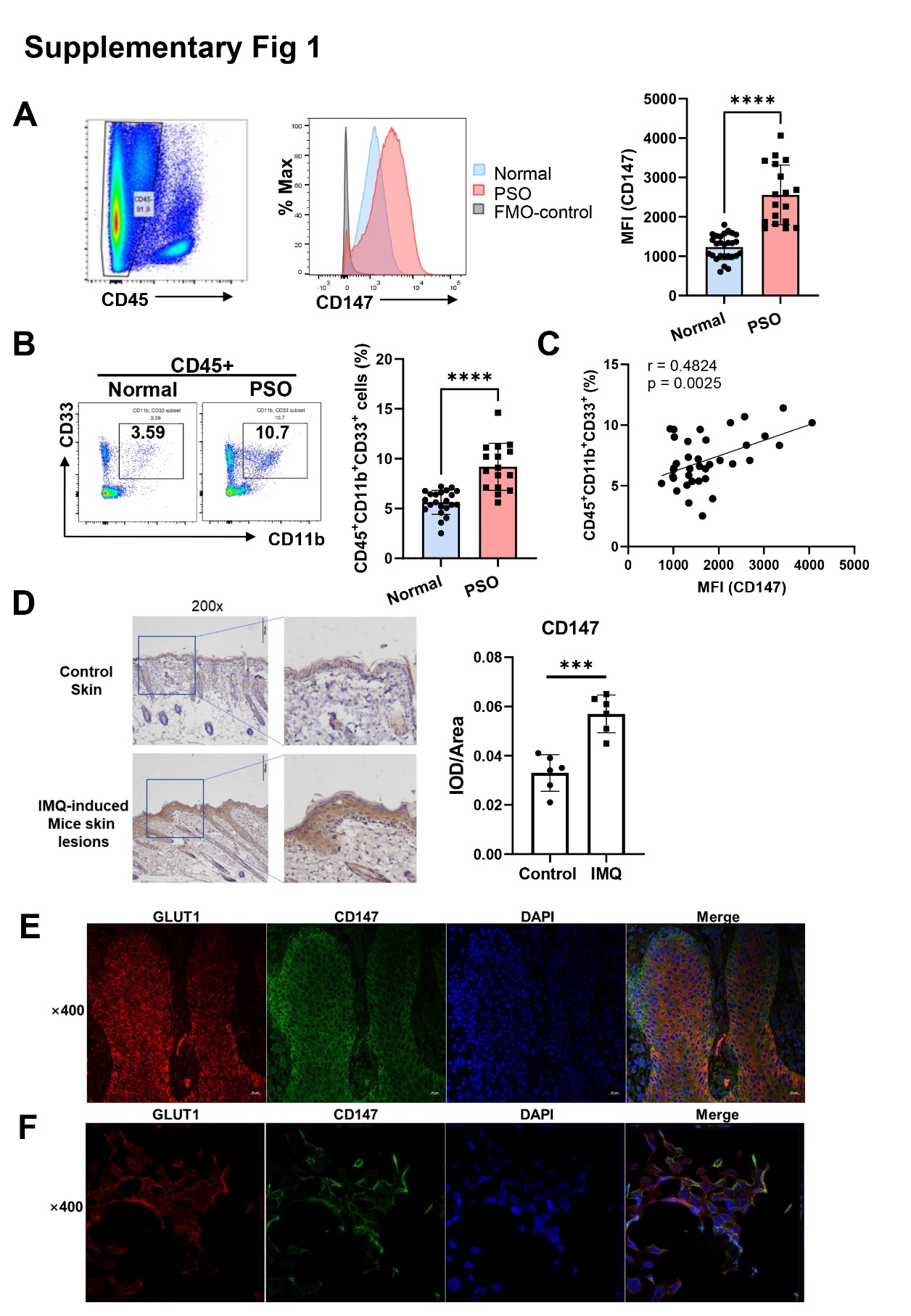


Fig S1. The abundance of epidermal CD147 in skin lesions of patients with psoriasis is related to inflammatory cell infiltration. (**A**) Representative flow cytometry panels for quantification of CD45^-^CD147^+^ cells. Statistical analysis of MFI(CD147) between two groups is shown in the right panel. (**B**) Representative flow cytometry panels for quantification of CD45^+^CD11b^+^CD33^+^ cells. Statistical analysis of those inflammatory cells (%) is shown in the right panel. (**C**) The positive correlation between CD45^+^CD11b^+^CD33^+^(%) and MFI(CD147). (**D**) IHC were conducted to detect the expression of CD147 in IMQ-induced skin lesions and control skins. (**E and F**) The co-localization of CD147 and GLUT1 on the cell membrane by using laser confocal microscopy (B, Psoriasis skin lesion; C, HaCaT cells). ****P < 0.0001, ***P < 0.001, ns, not significant. two-tailed unpaired Student’s t test was used.


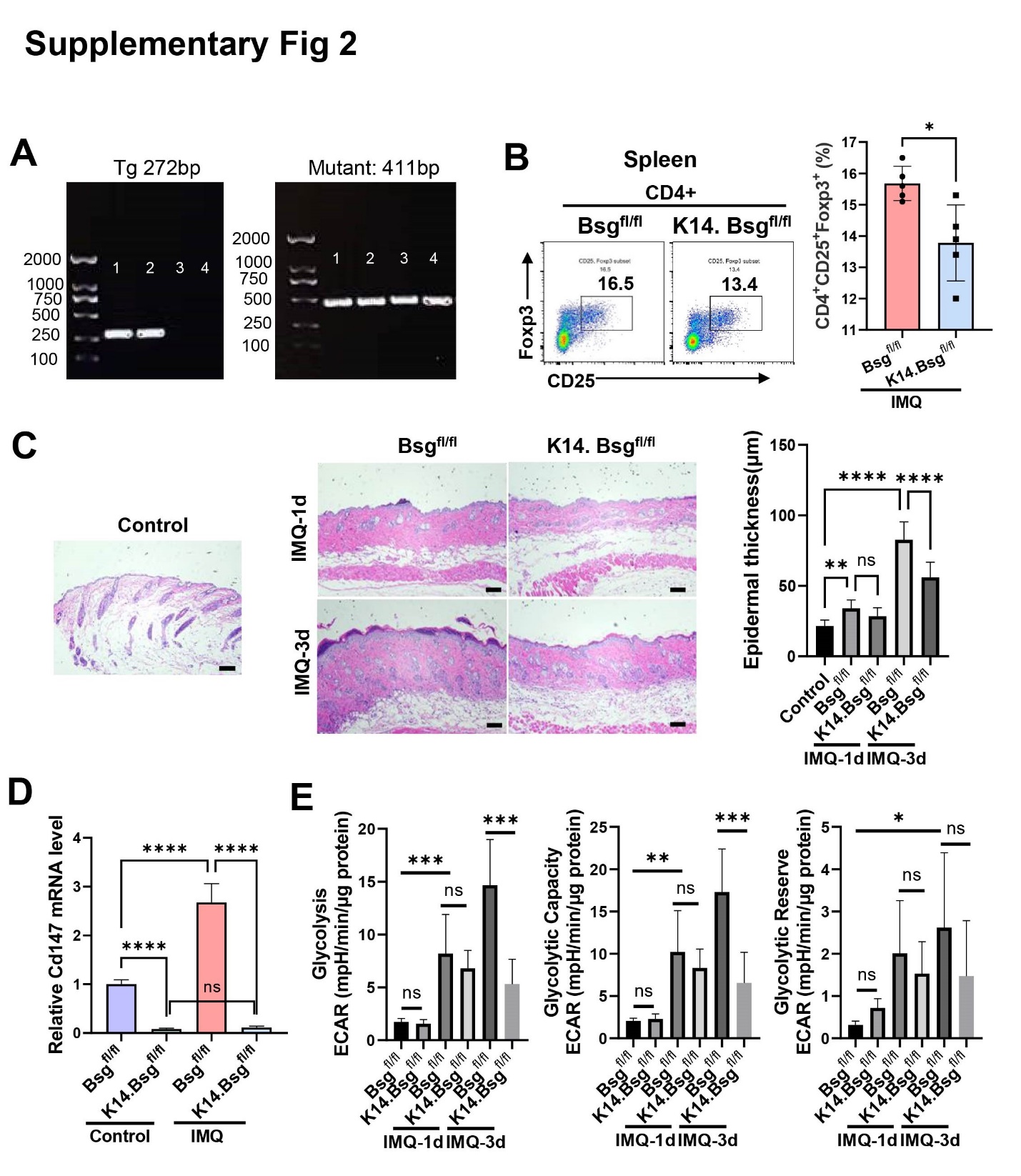


Fig S2. Depleting CD147 attenuates IMQ-induced psoriasis-like skin inflammation and down-regulates inflammatory cell infiltration. (**A**) Representative genotyping of K14.Bsg^fl/fl^ transgenic mice. Tg 272bp, Mutant 411bp. (**B**) Representative flow cytometry panels for quantification of Tregs in spleens of Bsg^fl/fl^ and K14.Bsg^fl/fl^ mice induced by IMQ. (**C**) The H&E staining of the dorsal skins derived from Bsg^fl/fl^ and K14.Bsg^fl/fl^ mice induced by IMQ for 0/1/3 days. (One representative mouse from each group is presented, n = 3-6 mice per group). Scale bars: 100 μm. Statistical analysis of epidermal thickness is shown in the right panel. (**D**) Relative mRNA expression of Cd147 in epidermis of Bsg^fl/fl^ and K14.Bsg^fl/fl^ mice induced by IMQ or not. (**E**) Specific data statistics of glycolysis, glycolysis capacity and glycolysis reserve level of Fig 2b. *P < 0.05, **P < 0.01, ***P < 0.001, ****P < 0.0001, ns, not significant. two-tailed unpaired Student’s t test or one-way ANOVA with Dunnett’s post hoc test was used.


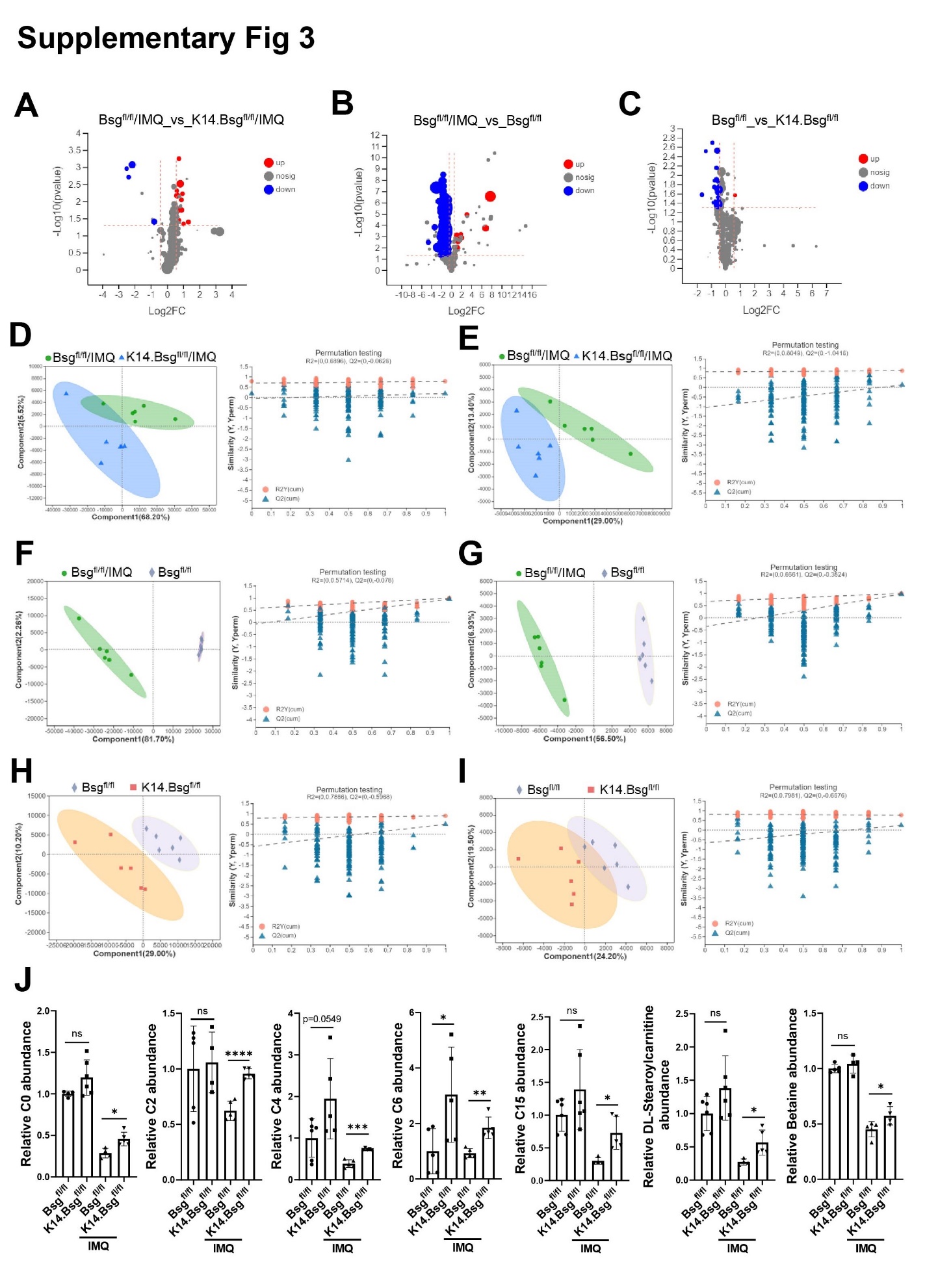


Fig S3. Non-targeted metabolomics profiling analysis for epidermis of Bsg^fl/fl^ and K14.Bsg^fl/fl^ mice. (**A–C**) Volcano plot of differentially abundant metabolites between the Bsg^fl/fl^/IMQ group and K14.Bsg^fl/fl^/IMQ group (**A**), the Bsg^fl/fl^/IMQ group and Bsg^fl/fl^ group (**B**), and the Bsg^fl/fl^ control group and K14.Bsg^fl/fl^ control group (**C**). (**D**), (**E**) PLS-DA score plots from the Bsg^fl/fl^/IMQ group and K14.Bsg^fl/fl^/IMQ group in (**D**) positive mode and (**A**) negative mode. (**F**), (**G**) PLS-DA score plots from the Bsg^fl/fl^/IMQ group and Bsg^fl/fl^ group in (**F**) positive mode and (**G**) negative mode. (**H**), (**I**) PLS-DA score plots from the control groups in (**H**) positive mode and (**I**) negative mode. The validation plots shown in the right panel were obtained from 200 permutation tests in positive mode and negative mode respectively. (**J**) The relative abundance of several differentially abundant metabolites. *P < 0.05, **P < 0.01, ***P < 0.001, ****P < 0.0001, ns, not significant. One-way ANOVA with Dunnett’s post hoc test was used.


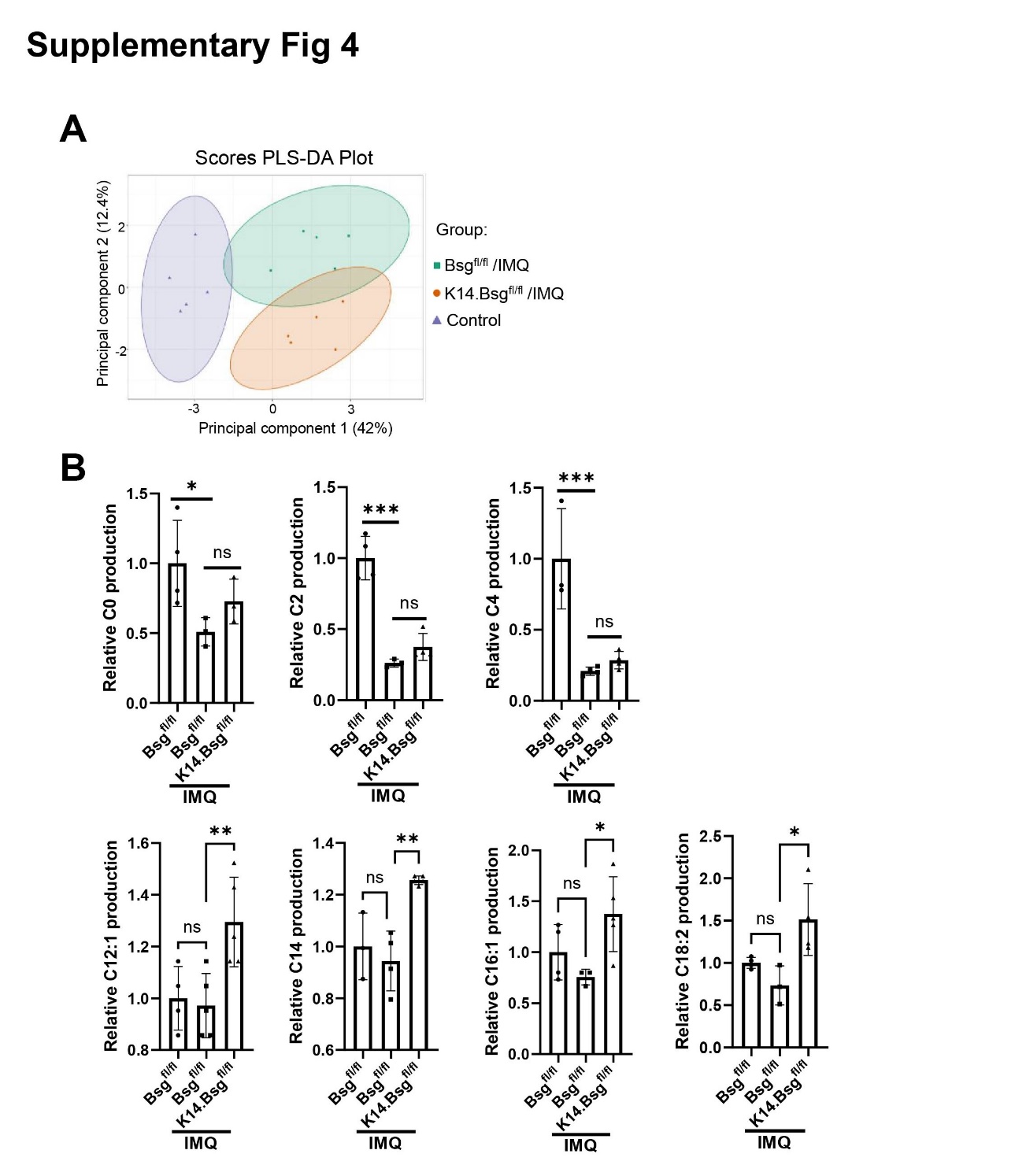


Fig S4. Carnitine-targeted metabolomics profiling analysis for epidermis of Bsg^fl/fl^ and K14.Bsg^fl/fl^ mice. (**A**) PLS-DA score plots among 3 groups. (R^2^X=0.544, R^2^Y=0.796, Q^2^=0.599, P < 0.005). (**B**) The relative concentration distribution of differential carnitine and acylcarnitines in IMQ-induced mouse models among 3 groups. *P < 0.05, **P < 0.01, ***P < 0.001, ****P < 0.0001, ns, not significant. One-way ANOVA with Dunnett’s post hoc test was used.


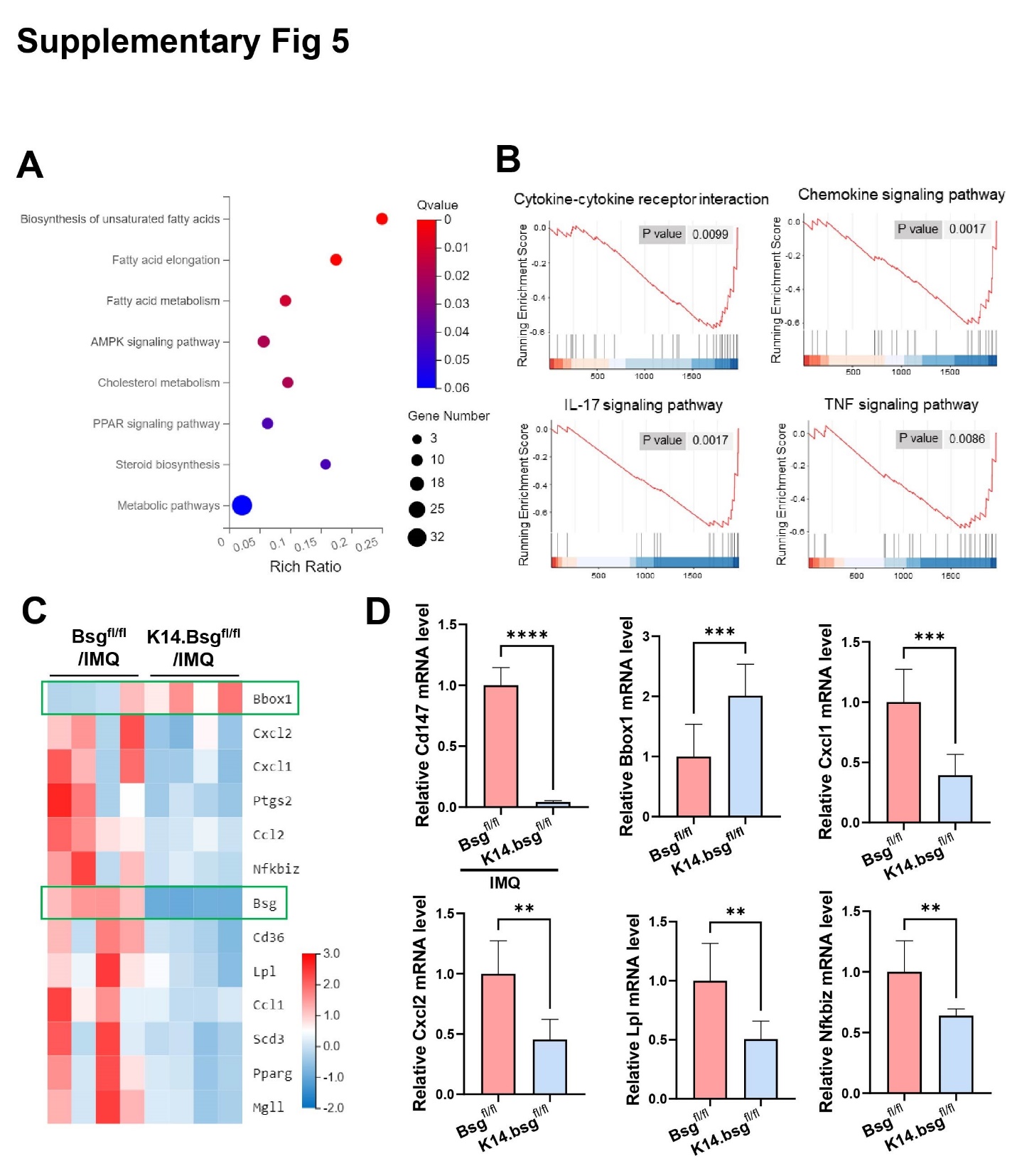


Fig S5. The effect of depletion of CD147 on gene expression profiles of epidermis of IMQ-induced mice. (**A**) The KEGG pathway showed the top significant function enriched pathway among differentially expressed genes. (**B**) GSEA enrichment plots for the immunologic signatures in K14.Bsg^fl/fl^ mice skin relative to Bsg^fl/fl^ mice skin. (**C**) Heatmap illustrates the expression levels of differentially expressed genes between the two groups. (**D**) Relative mRNA expression of the differentially screened genes (n = 3 – 6 mice per group). *P < 0.05, **P < 0.01, ***P < 0.001, ****P < 0.0001, ns, not significant. two-tailed unpaired Student’s t test was used.

| Table S1. Demographics of patients with psoriasis and healthy control subjects | | |
| --- | --- | --- |
| **Characteristics** | **Psoriasis Patients** | **Healthy Control subjects** |
| Number of analyzed patients | 18 | 27 |
| Age in years, mean ± SD | 34 ± 13 |  |
| Gender | 61% males, 39% females |  |
| Race/ethnicity | 100% Chinese |  |
| PASI score, mean(range) ± SD | 4.4(0-10.5) ± 3 | N/A |
| Abbreviations: N/A, not applicable; PASI, Psoriasis Area and Severity Index. | | |

| Table S2. A list of primers used for qPCR | | | |
| --- | --- | --- | --- |
| **Gene** | **Species** |  | **Primer sequence** |
| Cxcl1 | mice | Forward | GCAGACCATGGCTGGGATTC |
|  |  | Reverse | AAGCCTCGCGACCATTCTTG |
| Cxcl2 | mice | Forward | AAAATCATCCAAAAGATACTGAACAA |
|  |  | Reverse | CTTTGGTTCTTCCGTTGAGG |
| Il-6 | mice | Forward | GCTACCAAACTGGATATAATCAGGA |
|  |  | Reverse | CCAGGTAGCTATGGTACTCCAGAA |
| Il-1α | mice | Forward | CGAAGACTACAGTTCTGCCATT |
|  |  | Reverse | GACGTTTCAGAGGTTCTCAGAG |
| Il-1β | mice | Forward | GCAACTGTTCCTGAACTCAACT |
|  |  | Reverse | ATCTTTTGGGGTCCGTCAACT |
| VEGF | mice | Forward | AAAAACGAAAGCGCAAGAAA |
|  |  | Reverse | TTTCTCCGCTCTGAACAAGG |
| S100A8 | mice | Forward | TCCTTGCGATGGTGATAAAA |
|  |  | Reverse | GGCCAGAAGCTCTGCTACTC |
| S100A9 | mice | Forward | CACAGTTGGCAACCTTTATGAA |
|  |  | Reverse | TCATACACTCCTCAAAGCTCAG |
| Cd147 | mice | Forward | GCACTACTCGGGAAACCATCTCAC |
|  |  | Reverse | ACCAGGACCTCAGCCACGATG |
| Ptgs2 | mice | Forward | GGTGCCTGGTCTGATGATGTATGC |
|  |  | Reverse | CCTATGAGTATGAGTCTGCTGGTTTGG |
| Lpl | mice | Forward | CGCTCTCAGATGCCCTACAAAGTG |
|  |  | Reverse | TTGTGTTGCTTGCCATCCTCAGTC |
| Nfkbiz | mice | Forward | TCTCACTTCGTGACATCACC |
|  |  | Reverse | GGTTGGTATTTCTGAGGTGGAG |
| Bbox1 | mice | Forward | ATGGGGCTCATTTGATGCAGA |
|  |  | Reverse | GAAGTTTCCGAGCTTTTGCAG |
| Slc2a1 | mice | Forward | GAAGAAGGTCACCATCTTGGAG |
|  |  | Reverse | CGAAGATGCTCGTTGAGTAGTA |
| Hk1 | mice | Forward | GCTACATGGAGGAACTGCGACAC |
|  |  | Reverse | GTACATGCCGCTCACCATCTTCTC |
| Hk2 | mice | Forward | GCGTGGATGGCTCTGTCTACAAG |
|  |  | Reverse | GGAGGAAGCGGACATCACAATCG |
| Hk3 | mice | Forward | GCCGCACCTGTGTCAGCATC |
|  |  | Reverse | TGGACCAGAACCAGCCTCACC |
| Pkm | mice | Forward | GTGCCGCCTGGACATTGACTC |
|  |  | Reverse | TTCAGCCGAGCCACATTCATTCC |
| Ldhb | mice | Forward | TGGTGGATGTGTTGGAAGACAAGC |
|  |  | Reverse | CTCCTGCTGGCGGACTCCTG |
| β-actin | mice | Forward | GCTCTGGCTCCTAGCACCAT |
|  |  | Reverse | GCCACCGATCCACACAGAGT |

| Table S3. A list of primers for used ChIP-qPCR | | | |
| --- | --- | --- | --- |
| **Gene** | **Product**  **Length** |  | **Primer sequence** |
| Bbox1 Primer1  (-136bp, +264bp) | 400 | Forward | AGCTTCCAGGAGAAGTTTCCG |
|  |  | Reverse | ACAGCTAGTCACTGTGCTTCT |
| Bbox1 Primer2  (-424bp, -41bp) | 384 | Forward | CTCAGCAGTGTGAGCCTACAG |
|  |  | Reverse | CCAGATGCCTCTGTCATTGGT |
| Bbox1 Primer3  (-1136bp, -782bp) | 355 | Forward | AGGCACCAGGGTACTTAGACT |
|  |  | Reverse | TTTGCTTTGCTTGGTCGGTC |
| Bbox1 Primer4  (-1546bp, -1101bp) | 446 | Forward | CTGAAACAGCACCAGGGACC |
|  |  | Reverse | ACTTTTGCTCAATGCCCAAGC |

| Table S4. A list of antibodies used for flow cytometry | | | |
| --- | --- | --- | --- |
| **For mice** | **Source** | **For human** | **Source** |
| Zombie Aqua™ Fixable Viability Dye | BioLegend | APC/Cy7 anti-human CD45 | BioLegend |
| Trustain fcX anti-mouse CD16/32 | BioLegend | PerCP/Cy 5.5 anti-human CD11b | BioLegend |
| APC/Cy7 anti-mouse CD45 | BioLegend | APC anti-human CD33 | BioLegend |
| PE/Cy7 anti-mouse/human CD11b | BioLegend | FITC anti-human CD147 | BioLegend |
| PerCP/Cy 5.5 anti-mouse Gr-1 | BioLegend |  |  |
| PE Rat anti-mouse IL-17A | BD Pharmingen |  |  |
| APC anti-mouse IFN-r | BioLegend |  |  |
| BV421 anti-mouse CD4 | BioLegend |  |  |
| Anti-mouse CD25 APC | BioLegend |  |  |
| Anti-mouse/rat Foxp3 PE | eBioscience |  |  |
